# Comparison of Peri-Implant Soft Tissue Around Zirconia and Titanium Abutments in the Aesthetic Zone: A Narrative Review

**DOI:** 10.7759/cureus.65782

**Published:** 2024-07-30

**Authors:** Turki M Abu Al-Faraj, Bashaer M Alsubhi, Abeer N Almarhoon, Abdulaziz A Almarshoud, Mohammed S Alqattan, Shahad H Alqahtani, Ahmed A Al Osaimi, Latifah Saad Alshammari, Abdullah I Almakrami, Yazeed S Alwadai

**Affiliations:** 1 Dentistry, National Guard Hospital, Madinah, SAU; 2 Dentistry, Ibn Sina National College, Jeddah, SAU; 3 Dentistry, King Abdulaziz University, Jeddah, SAU; 4 Dentistry, Imam Abdulrahman Bin Faisal University, Dammam, SAU; 5 Dentistry, Al-Tabeeb Clinic, Riyadh, SAU; 6 Dentistry, Zahur Al-Qarat Medical Company, Riyadh, SAU; 7 Dentistry, University of Hail, Hail, SAU; 8 Dentistry, Najran University, Najran, SAU; 9 Dentistry, Armed Forces Hospital, Jubail, SAU

**Keywords:** aesthetic zone, titanium abutments, zirconia abutments, maxillary anterior zone, peri-implant soft tissue

## Abstract

This narrative review compares the peri-implant soft tissue responses around zirconia and titanium abutments in the aesthetic zone, emphasizing their mechanical, biological, and aesthetic properties. Titanium abutments, known for their excellent mechanical strength and fatigue resistance, have traditionally been the standard in dental restorations but face challenges in aesthetic integration due to their metallic appearance and potential for higher inflammatory responses. Zirconia abutments, emerging as a promising alternative, offer superior aesthetic outcomes, reduced plaque accumulation, and lower inflammatory responses, making them ideal for use in visible areas with thin soft tissue biotypes. However, zirconia's mechanical properties, such as lower fracture resistance, necessitate careful clinical application. The review also highlights rare instances of titanium allergies, underscoring the importance of individualized treatment planning and regular monitoring to ensure the longevity and success of implant restorations.

## Introduction and background

Replacement of missing teeth by dental implants in complete and partial edentulism is a widely accepted treatment modality. The maxillary anterior zone is a critical zone from both the surgical and prosthetic point of view. Detailed planning, prosthetically driven precise implant placement, soft tissue analysis, and appropriate material selection for the abutment and final crown are essential to get the best treatment outcome from an aesthetic point of view. The implant abutment, which connects the intraosseous implant to the overlying implant restoration, is an important prosthetic component. Titanium abutments have been a gold standard for implant-supported restorations in all areas of the mouth. However, in patients with thin, soft tissues, a dark halo around the abutments can be seen. The metal abutment interferes with the reflection of light, leading to a darkish/grayish appearance [1]. To counteract this problem, different materials have been used in place of titanium, such as gold, various coatings on titanium abutments, alumina, and zirconia [2].

As the aesthetic requirements of patients have increased along with advances in dental material science, the use of zirconia as an abutment material has gradually risen over the last decade [3]. The mechanical properties of zirconia are superior to any other ceramic material due to its stress-induced transformation toughening [4]. In areas of thin gingival tissue, zirconia abutments have shown a favorable color response and better aesthetic scores as measured by pink aesthetic scores (PES) [1,5].

The peri-implant soft tissue is similar to the natural periodontium, with more similarity to scar tissue in terms of collagen, vascularity, and composition. The peri-implant epithelium is attached to the implant by hemidesmosomes, but their presence has been disputed [6,7]. A strong seal of the soft tissue with the implant is the first line of defense against bacterial pathogens. Studies have reported the incidence of pocket formation due to epithelial downgrowth at the peri-implant interface to be higher than in natural teeth [8]. These pockets harbor anaerobic bacteria, which cannot be mechanically cleaned, leading to inflammation and, ultimately, bone destruction. A strong seal of the implant-abutment junction with the soft tissues would prevent this bacterial ingress and further complications. The aim of this review is to compare the peri-implant soft tissue responses and clinical outcomes of zirconia and titanium abutments in the aesthetic zone. By evaluating their mechanical, biological, and aesthetic properties, the review aims to inform dental practitioners on the best material choice for optimizing implant success and aesthetics, with considerations for potential titanium allergies and personalized treatment planning.

## Review

Research methodology

A comprehensive search was conducted on digitized databases, including PubMed, Embase, Medline, and Google Scholar, utilizing specific keywords such as "zirconia abutments," "titanium abutments," "peri-implant soft tissue," "aesthetic zone," and "dental implants." The initial screening involved identifying relevant articles through title and abstract review, followed by a full-text evaluation of the selected studies.

The inclusion criteria for this review comprised narrative reviews, systematic reviews, randomized controlled trials, comparative studies, clinical trials, in vitro studies, and case series focusing on the comparison between zirconia and titanium abutments in terms of mechanical, biological, and aesthetic outcomes. Studies that examined peri-implant soft tissue response, implant success rates, and patient satisfaction were also included.

Conversely, studies that focused on surgical interventions, the use of antimicrobial therapies, or abutment materials other than zirconia and titanium were excluded. Additionally, articles that did not provide specific data on the maxillary anterior region or those lacking sufficient methodological detail were omitted from this review.

Peri-implant soft tissue in the maxillary anterior zone

The peri-implant soft tissue is comprised of epithelium and connective tissue. This tissue is formed as a result of wound healing [9]. The internal basal lamina and the hemidesmosomes attach the epithelium part to the implant. Atsuta et al. [10] found that compared to natural teeth, the epithelial seal around dental implants is significantly less due to the lower number of hemidesmosomes and internal basal lamina. The authors also said that these structures are concentrated more in the lower part of the implant-peri-implant epithelium surface, thereby leading to lower strength of the attachment assembly. Another reason for the lower strength is the time required for the maturation of the assembly. As per literature reports, seven days are required for the initial attachment, and another two to four weeks are required for the final maturation [9,11,12]. The connective tissue consists of gingival and periodontal ligament (PDL) fibers. Around dental implants, the fibers are arranged in a parallel fashion, and physical adaptation of the fibers is noted without any attachment or biological integration [13]. Al Rezk et al. [14] noted that the percentage of gingival fibers is significantly less (only 3%-5%) than around natural teeth. The reduced number of fibers also leads to a longer post-maturation time of the attachment assembly.

Atsuta et al. [10] stated that the peri-implant soft tissue has no blood supply of its own and is dependent on the surrounding periosteum and the connective tissue for nourishment. In the maxillary anterior region, where the gingiva is thin, it is advocated that the implant be placed 2 mm cervical to the proposed gingival margin to compensate for the gingival recession that may happen post-placement. Additionally, free gingival grafts or subepithelial connective tissue grafts are recommended to maintain the periosteum and improve the blood flow to the area, thereby maintaining the soft tissue level/attached gingiva around the implant and preventing gingival recession. An average of 2 mm of keratinized gingiva and 1 mm of attached mucosa are essential around any implant to provide function and aesthetics [15]. The keratinized mucosa helps prevent recession around the implant sites, thereby preventing angular bone loss around the implant and, thereby, peri-implantitis. It is also important to check for the gingival biotype in the maxillary anterior region. Two types of biotypes have been identified: thin and thick [16]. Gingival recession is expected in patients with a thin biotype. Also, the underlying color of the implant abutment may be visible in thin biotype patients. The thick biotype has a greater tendency for pocket formation and scar tissue in sites of the releasing incisions.

Understanding the biological and structural properties of peri-implant soft tissue is crucial for optimizing implant success in the anterior maxillary zone. With the foundational knowledge of how soft tissues interact with implants, we now turn our attention to the materials used in the abutments themselves. Titanium abutments have been the standard for many years, providing reliable mechanical properties but facing challenges in aesthetic integration. The development of surface modifications, such as titanium nitride (TiN) coatings, marks a significant advancement in addressing these challenges, particularly in the highly visible anterior region.

Titanium abutments in the anterior region

For many years, standardized titanium abutments provided by manufacturers were the only option available for dental practitioners to restore missing teeth in the anterior zone. While these prefabricated components simplified the technical aspects of dental restoration, they often fell short in terms of aesthetics. Several surface modifications and coatings for titanium abutments have been developed and reviewed to address this limitation. Among these, titanium nitride (TiN) coatings have shown significant promise in enhancing the aesthetic, biocompatible, and mechanical properties of titanium abutments, making them particularly suitable for use in the highly visible anterior region.

Surface coatings and their impact on aesthetic and bacterial resistance

Titanium nitride (TiN) coatings significantly enhance the aesthetic, biological, and mechanical properties of titanium abutments. According to Areid et al. [17], TiN coatings impart a gold-like appearance to the abutments, which improves visual integration with natural teeth and maintains aesthetic quality over time. This is particularly beneficial for anterior restorations where the visibility of the abutment is high. This gold-like appearance, due to a natural phenomenon known as light interference, enhances the visual integration of the abutment, particularly in the anterior aesthetic zone. This gold color can be advantageous in providing a more natural-looking appearance under thin gingival tissues, which is crucial for achieving desirable aesthetic outcomes in visible areas [2].

In terms of biocompatibility, TiN coatings support the adhesion, proliferation, and migration of gingival epithelial cells, including human gingival epithelial keratinocytes, which are crucial for forming a stable epithelial attachment and biological seal around the implant, thus preventing bacterial invasion and peri-implantitis [17,18]. Additionally, TiN coatings exhibit lower friction and enhanced corrosion resistance, which reduces inflammatory responses and metallic ion release, thereby promoting healthier soft tissue interactions [18]. TiN coatings improve the surface properties of titanium abutments, maintaining their biocompatibility while modifying the surface to reduce bacterial colonization and biofilm formation. Studies have shown that TiN coatings enhance fibroblast cell proliferation, attachment, and differentiation, which are essential for soft tissue health around the implant [2].

Mechanically, TiN coatings enhance the hardness and wear resistance of titanium abutments, ensuring durability and longevity. These coatings can increase the hardness of the titanium surface by approximately 10 times, providing better abrasion resistance and longevity. This makes the abutments more durable and less susceptible to mechanical wear during clinical use. The combination of enhanced mechanical properties with the ability to withstand the oral environment's stresses ensures the long-term success of the implant restorations [17]. It was noted by del Castillo et al. [19] that TiN coatings provide a robust protective layer that mitigates wear and abrasion, ensuring the mechanical stability of the implant-abutment connection over time. Therefore, TiN-coated titanium implant abutments offer significant advantages in aesthetics, biocompatibility, and mechanical properties, making them an excellent choice for achieving superior clinical outcomes in dental implantology [19].

Zirconia abutments in the anterior region

Following the advancements in titanium abutment coatings, particularly TiN, the dental field has continued to explore and develop alternative materials that can offer superior aesthetics and biological compatibility. One such material that has gained considerable attention is zirconia. The emergence of zirconia as an abutment material in the maxillary anterior region is driven by the desire for lifelike restorations and the need to address some of the limitations associated with titanium abutments.

Clinical outcomes and recommendations

Several studies have demonstrated the efficacy of zirconia implant abutments. Wittneben et al. [20] conducted a five-year study comparing prefabricated zirconia abutments with computer-aided design/computer-aided manufacturing (CAD/CAM) zirconia abutments in the anterior maxilla. Group A (prefabricated) had a 95% survival rate, while group B (CAD/CAM) had a 90% survival rate. Both groups showed stable bone levels and aesthetic outcomes. Prefabricated abutments are simpler, while CAD/CAM abutments offer tailored solutions for individual anatomy and aesthetic needs.

Passos et al. [21] assessed the performance of zirconia abutments over 12 years, finding that standard platform implants exhibited higher marginal bone loss compared to platform-switching designs for up to five years. Survival rates were 93.8% for standard implants and 90% for platform-switching implants. The study highlighted the durability of zirconia abutments and suggested that platform switching may better preserve bone levels despite a higher fracture risk. Chen et al. [22] reported a 100% survival rate for zirconia abutments and crowns over six years, with excellent aesthetic outcomes and minimal bone loss. These findings reinforce zirconia's suitability for anterior and premolar restorations, offering superior aesthetic integration and biological compatibility.

Several clinical recommendations can be made for the usage of zirconia abutments in the maxillary anterior region. Naveau et al. [23] recommend zirconia abutments with titanium inserts for enhanced fracture resistance. Caution is advised for angulations exceeding 20-30 degrees to prevent fractures. CAD-CAM custom abutments offer better soft tissue stability, particularly in the anterior region. De Holanda Cavalcanti Pereira et al. [24] emphasized ensuring a minimum wall thickness of 0.8 mm to prevent fractures. Gou et al. [25] stressed avoiding overpreparation and overload of zirconia abutments. Chen et al. [22] highlighted the importance of regular monitoring and maintenance to detect early signs of wear or misfit.

Overall, zirconia abutments offer substantial advantages in survival, aesthetics, and biological outcomes. Individualized treatment planning and careful case selection are crucial to optimize outcomes. Regular monitoring and maintenance ensure the longevity and success of zirconia abutments in dental implantology.

Comparison of titanium with zirconia abutments

Following advancements in titanium abutment coatings and the exploration of zirconia as an alternative material, the dental field has extensively studied the mechanical, biological, and aesthetic properties of titanium and zirconia abutments. This section compares these materials to highlight their respective strengths and weaknesses in clinical applications.

Mechanical considerations: zirconia versus titanium abutments

In a comprehensive evaluation of zirconia abutments, several studies have detailed their mechanical properties and clinical performance. In a systematic review by Naveau et al. [23], the mechanical properties of zirconia abutments in the anterior region were thoroughly examined, analyzing 20 studies with a minimum of one-year follow-up. The findings revealed a fracture rate between 1.2% and 8%, with screw loosening observed in up to 6% of cases, but no instances of chipping. Additionally, zirconia abutments with titanium inserts were highlighted as a promising design for enhancing mechanical strength, although data on their clinical performance remain limited. Despite their excellent aesthetic performance, the review emphasized the mechanical risks associated with zirconia abutments, particularly their propensity for fractures, underscoring the importance of careful clinical application to minimize these risks [23]. A systematic review by de Holanda Cavalcanti Pereira et al. [24] compared the mechanical properties of zirconia and titanium abutments, focusing on wear and misfit at the implant-abutment interface. The review included nine in vitro studies, analyzing 172 specimens (86 zirconia and 86 titanium abutments). Zirconia abutments caused more severe wear, more scratches, and rounding of the hexagonal angles at the implant connection interface than titanium abutments. Zirconia abutments also exhibited a greater degree of misfit at the connection interface, while titanium abutments demonstrated a better fit, influenced by the manufacturing method [24].

According to Carrillo de Albornoz et al. [26], titanium abutments are renowned for their excellent mechanical properties, making them a popular choice in dental applications. They exhibit high tensile and compressive strength, ensuring durability and reliability under functional loads. Titanium's ductility allows it to deform under stress without breaking, accommodating minor misalignments and reducing the risk of catastrophic failure. Additionally, titanium's superior fatigue resistance enables it to withstand the cyclic loading experienced in the oral environment over prolonged periods, further contributing to its longevity. Halim et al. [27] highlighted that titanium abutments demonstrate higher resistance to fracture and greater flexural strength than zirconia abutments. The inherent brittleness and lower fracture resistance of ceramic materials such as zirconia contribute to a higher incidence of abutment fractures, which can vary from 1.08% to 17.86%. Factors influencing these rates include patient age, gender, tooth position, and implant-abutment connection design. Zirconia abutments with one-piece internal connections tend to have higher fracture rates compared to external and two-piece internal connections, particularly in posterior areas, due to higher occlusal forces [27].

Queiroz et al. [28] noted that the modulus of elasticity is another critical factor when comparing these materials. Titanium's lower modulus of elasticity indicates greater flexibility, allowing it to absorb and distribute masticatory forces more effectively, reducing stress transferred to the bone-implant interface. In contrast, zirconia's higher modulus of elasticity makes it more rigid, transferring more stress directly to the surrounding bone and implant, potentially leading to complications if not managed properly. While zirconia's superior wear resistance ensures long-term surface integrity, making it suitable for aesthetic areas, titanium's wear resistance, although adequate, is not as high and can exhibit wear when in contact with harder materials [28].

Overall, the choice between titanium and zirconia abutments in the anterior teeth area often depends on specific clinical requirements. Titanium abutments offer superior mechanical properties, including strength and fatigue resistance, making them a reliable choice for situations requiring durability and longevity. Conversely, zirconia abutments provide excellent wear resistance, making them suitable for areas where mechanical performance is critical. The decision should be tailored to the individual patient's needs, balancing mechanical performance to achieve optimal results in the anterior teeth area.

Biological considerations

Zirconia abutments have demonstrated several favorable biological properties compared to titanium abutments. Sanz-Martín et al. [29] observed that zirconia abutments experienced less increase in bleeding on probing (BOP) values over time and less plaque accumulation than titanium abutments. This reduced plaque accumulation on zirconia is attributed to its surface properties, resulting in less microbial colonization and reduced inflammation. Although histological studies comparing soft tissue response to zirconia and titanium found similar soft tissue dimensions around both materials, indicating comparable biological integration, titanium abutments tend to accumulate more plaque and result in higher BOP values, potentially leading to greater inflammation and peri-implant diseases.

Zirconia abutments offer significant advantages in soft tissue integration. Tang et al. [30] highlighted that stable soft tissue integration around zirconia abutments is crucial for preventing pathogen penetration, protecting underlying bone tissue, and preventing peri-implantitis. This stability is particularly important for maintaining long-term implant stability, especially in the anterior region and for patients with thin gingival biotypes. Surface treatment methods, such as micro- and macrostructural designs, aim to enhance soft tissue adhesion to zirconia abutments by promoting fibroblast viability, adhesion, and proliferation. This results in a stronger mucosal seal, better healing of surrounding soft tissues, and higher soft tissue integrity compared to titanium abutments. Additionally, zirconia's lower inflammatory infiltration and reduced bacterial adhesion make it a favorable choice for maintaining peri-implant health.

Barwacz et al. [31] found that titanium abutments demonstrated significantly elevated levels of leptin compared to zirconia abutments, suggesting a biomaterial-specific regulation of peri-implant mucosa or bone metabolism. Elevated leptin levels indicate a distinct biological response, potentially contributing to a higher inflammatory response around titanium abutments. In contrast, zirconia abutments maintain stable levels of pro-inflammatory cytokines and bone metabolism mediators, supporting their favorable biocompatibility profile. The lower inflammatory response associated with zirconia abutments may contribute to their increased preference in clinical settings, particularly for patients with high aesthetic demands or thin gingival biotypes. Nogueira-Filho et al. [32] conducted a longitudinal study comparing cytokine levels in peri-implant crevicular fluid (PICF) and gingival crevicular fluid (GCF) in healthy patients. The study revealed that titanium abutments exhibit pro-inflammatory responses due to bacterial biofilm colonization, stimulating the secretion of cytokines such as IL-1β and TNF-α, which are associated with tissue degradation and bone loss around implants. In contrast, zirconia abutments demonstrated lower levels of pro-inflammatory cytokines in PICF, suggesting a reduced likelihood of provoking an inflammatory response, which is beneficial for long-term peri-implant health.

Gehrke et al. [33] emphasized that both titanium and zirconia abutments are highly biocompatible but interact differently with soft tissues. Titanium forms a stable oxide layer that prevents corrosion and promotes osseointegration, ensuring excellent biocompatibility. However, zirconia's biocompatibility, combined with its tooth-colored appearance, provides an aesthetic advantage, particularly in the anterior region, minimizing the risk of gingival darkening that can be a concern with titanium abutments in cases of thin gingival biotype.

Aesthetic considerations

The aesthetic evaluation of peri-implant soft tissue around zirconia and titanium abutments reveals notable differences. Zirconia abutments tend to offer superior aesthetic outcomes compared to titanium abutments, particularly in terms of soft tissue color and overall appearance. Studies have shown that zirconia abutments result in a better color match with the surrounding gingiva, reducing the likelihood of a grayish hue that can be evident with titanium abutments due to their metallic nature. This is particularly significant in patients with thin peri-implant mucosa, where the darker shade of titanium can be more noticeable and less aesthetically pleasing [34].

Moreover, zirconia abutments have been associated with higher pink esthetic scores, reflecting better integration with the natural color and texture of the gingiva. In a meta-analysis, zirconia abutments demonstrated significantly better color outcomes than titanium, which supports the preference for zirconia in aesthetically demanding situations [34]. Although there are no substantial differences between the two materials regarding biological parameters such as soft tissue recession and bleeding on probing, the aesthetic advantages of zirconia make it a preferred choice in the aesthetic zone [34]. Long-term studies also support the aesthetic superiority of zirconia abutments. In a randomized controlled clinical trial with a follow-up of at least seven years, zirconia abutments consistently outperformed titanium abutments in terms of aesthetic outcomes [1]. The study assessed 26 single-tooth implant-supported prostheses and found that zirconia abutments yielded higher pink aesthetic scores at all time intervals. Specifically, zirconia abutments showed mean pink esthetic scores of 8.25 ± 1.03 at the seven-year mark, compared to 7.44 ± 1.81 for titanium abutments [1]. Additionally, the white aesthetic score was higher for zirconia, reflecting a superior match with the natural tooth color and texture [1].

A randomized controlled clinical trial using a crossover design compared the aesthetic outcomes of different abutment materials in the anterior maxilla. The study involved 25 patients, each receiving three types of abutments: gray titanium, pink anodized titanium, and hybrid zirconia. The aesthetic evaluation was conducted using spectrophotometry to measure the color difference (ΔE) between the peri-implant mucosa and the contralateral natural teeth. The results indicated that zirconia abutments provided the closest color match to natural teeth, thereby offering superior aesthetic outcomes [35]. The pink esthetic scores were also highest for zirconia abutments (10.88 ± 0.88), with patient satisfaction measured using visual analog scales being higher for pink anodized and zirconia hybrid abutments compared to gray titanium abutments [35]. In a five-year randomized controlled trial comparing zirconia-based versus metal-based implant-supported single-tooth restorations in the premolar region, the aesthetic outcomes were meticulously evaluated. The study included 30 patients and found that both types of restorations yielded successful aesthetic outcomes, with no significant differences in professional-reported aesthetic scores between the all-ceramic crowns on zirconia abutments and metal-ceramic crowns on metal abutments [36]. Despite the differences in material properties, the zirconia abutments did not demonstrate a statistically significant aesthetic advantage over titanium abutments in this trial, indicating that both materials can be effectively used in the aesthetic zone for single-tooth restorations in the premolar region [36].

A systematic review of systematic reviews compared the clinical outcomes of titanium and zirconia implant abutments, highlighting their aesthetic impact on peri-implant soft tissues. The study included 11 systematic reviews and demonstrated that zirconia abutments generally offered better aesthetic outcomes compared to titanium abutments due to zirconia's toothlike color [27]. This advantage was more pronounced in patients with peri-implant soft tissue thickness less than 3 mm, where the underlying abutment material could more easily affect the visual outcome [27]. The pink esthetic score was consistently higher for zirconia abutments, indicating better soft tissue compatibility and a more natural appearance [27]. In a multicentric prospective study, the influence of abutment material on peri-implant soft tissue color in anterior areas with a thin gingival biotype was analyzed using spectrophotometric digital technology. This study evaluated gold, titanium, and zirconia abutments in the anterior maxilla, finding that gold and zirconia abutments provided a closer color match to the natural gingiva compared to titanium abutments [37]. For patients with facial soft tissue thickness of ≤2 mm, the ΔE mean values of gold and zirconia abutments were significantly closer to the critical threshold for intraoral color distinction by the naked eye, emphasizing the importance of abutment material selection in achieving optimal aesthetic outcomes in patients with thin gingival biotypes [37]. In a 16-year prospective clinical study comparing metal-ceramic and all-ceramic single crowns for implant-prosthetic rehabilitation of maxillary lateral incisor agenesis, both Auro Galvan Crowns and all-ceramic crowns provided satisfactory long-term results, with minor complications such as mucositis and decementation not significantly affecting the overall aesthetic success. However, patients with thin mucosal biotypes exhibited better aesthetic outcomes with zirconia abutments due to their superior color matching and translucency [38].

Considering the extensive evidence presented, it is clear that zirconia abutments consistently provide superior aesthetic outcomes compared to titanium abutments. This advantage is particularly evident in patients with thin peri-implant mucosa, where zirconia's toothlike color and light-diffusing properties prevent the unattractive grayish hue often associated with titanium. Although titanium abutments may offer comparable biological performance, the aesthetic benefits of zirconia are particularly valuable in the anterior region and other areas where visual appearance is paramount.

In conclusion, zirconia abutments offer significant aesthetic advantages over titanium abutments, particularly in the aesthetic zone and for patients with thin gingival biotypes. The superior color matching and higher pink esthetic scores associated with zirconia abutments make them the preferred choice for achieving natural-looking and pleasing peri-implant soft tissues. While titanium abutments remain a viable option due to their mechanical strength and biological compatibility, zirconia's ability to blend seamlessly with the surrounding gingiva and maintain aesthetic integrity over time underscores its importance in modern implantology. Clinicians should consider these factors when selecting abutment materials to ensure optimal aesthetic and functional outcomes for their patients. Table 1 shows a comparison between titanium and zirconium implants.

**Table 1:** Comparison of Aesthetic Outcomes: Zirconia Versus Titanium Abutments PES: pink esthetic score, WES: white esthetic score, ΔE values: color difference values

Citation Number	Aspect	Zirconia Abutments	Titanium Abutments
[1]	Color match with gingiva	Superior color match, less visible in thin gingival biotypes	Inferior color match, may show a grayish hue, especially in thin biotypes
[27]	Aesthetic score (PES/WES)	Higher PES and WES scores	Lower PES and WES scores
[34]	Overall aesthetic outcome	Reduces the likelihood of grayish hue, better color integration with gingiva	More likely to show a grayish hue, especially with thin gingiva
[35]	Peri-implant soft tissue color (ΔΕ values)	Lower ΔΕ values, closer color match to natural teeth	Higher ΔΕ values, less favorable color match
[36]	Long-term aesthetic outcomes	Provides satisfactory long-term aesthetic results, particularly beneficial for thin mucosal biotypes	Provides satisfactory long-term results but less favorable in thin mucosal biotypes
[37]	Peri-implant soft tissue color	Better color matching and integration of soft tissues, recommended for thin gingival biotypes	Greater likelihood of grayish appearance, especially in thin gingival biotypes
[38]	Aesthetic performance in the maxillary region	Better long-term aesthetic outcomes	Less favorable aesthetic outcomes in thin biotypes

Allergies to titanium

Recent clinical observations have underscored the complexity of managing patients with allergies to materials used in dental procedures, particularly titanium, which is widely used in implants and abutments. Titanium is widely acclaimed for its biocompatibility but has nevertheless been associated with allergic reactions in a subset of patients, raising concerns about its universal safety.

In an illustrative case reported by Lim et al. [39], a 70-year-old female experienced severe discomfort and mucosal inflammation around the TiN-coated titanium used in her dental procedure shortly after placement. Unlike areas with anodized titanium, the tissues surrounding the TiN-coated titanium exhibited significant redness and pain, confirmed through patch testing to be an allergic reaction. This led to the removal of the TiN-coated abutment and their replacement with uncoated titanium, which resolved the symptoms promptly within a week. This case highlights that even coatings intended to enhance the function and aesthetics of titanium can elicit allergic responses, which are crucial to identify and manage swiftly to restore patient comfort and tissue health.

Another compelling report by Hosoki et al. [40] detailed the case of a 69-year-old male who developed allergic contact dermatitis attributed to titanium used in his dental procedure. Initially, the patient had titanium screws placed for orthopedic reasons, which were suspected to have sensitized him to titanium. The subsequent exposure to titanium used in dental procedures exacerbated his condition, manifesting as persistent nummular eczema that only ameliorated following the removal of all titanium-containing dental materials. This case further emphasizes the potential for titanium to induce hypersensitivity reactions and the importance of considering prior exposures when evaluating patients for dental procedures. The review by Egusa et al. [41] described a particularly troubling case of a 50-year-old Japanese female who developed facial eczema following the placement of titanium dental materials. Despite the consensus on the biocompatibility of titanium, her allergic reaction persisted for two years, worsening her quality of life until the titanium was surgically removed. Subsequent improvement in her condition highlighted the critical role of comprehensive diagnostic evaluations, including patch testing and lymphocyte transformation tests, to ascertain the cause of such unusual dermatological reactions in patients with dental implants.

Collectively, these cases accentuate the nuanced challenges posed when using titanium in dental materials. While the incidence of titanium allergy is relatively low, its potential impact on patient health can be significant, necessitating vigilant patient evaluation and history taking. Clinicians should remain alert to the signs of metal hypersensitivity and consider alternative materials, such as zirconia, when faced with patients exhibiting or reporting allergic responses. The increasing use of titanium in various medical and dental applications only heightens the need for awareness and preparedness to manage such reactions effectively, ensuring that patient safety and comfort are not compromised. Table 2 shows a comparison between titanium and zirconia abutments.

**Table 2:** Comparison of Titanium and Zirconia Abutments BOP: bleeding on probing

Consideration	Titanium Abutments	Zirconia Abutments
Fracture rate	Lower fracture rate	Higher fracture rate
Wear and misfit	Better fit, influenced by manufacturing method	More severe wear, greater degree of misfit at connection interface
Mechanical properties	High tensile and compressive strength, superior fatigue resistance	Good mechanical properties but lower fracture resistance
Flexural strength	Higher resistance to fracture, greater flexural strength	Lower flexural strength, prone to fractures
Modulus of elasticity	Lower modulus of elasticity, more flexible	Higher modulus of elasticity, more rigid
BOP	Higher BOP values over time	Less increase in BOP values over time
Plaque accumulation	More plaque accumulation	Less plaque accumulation
Soft tissue integration	Stable soft tissue integration	Better soft tissue integration
Inflammatory response	Higher inflammatory response	Lower inflammatory response
Color match	Dark color, can cause a grayish appearance in thin biotypes	Better color match to natural soft tissue
Esthetic outcomes	Adequate esthetic outcomes	Superior esthetic outcomes, particularly in visible areas
Patient satisfaction	No significant difference in patient satisfaction	No significant difference in patient satisfaction
Allergic reactions	Potential for allergic reactions, although rare	No known allergic reactions

## Conclusions

The comparative analysis of peri-implant soft tissue around zirconia and titanium abutments in the aesthetic zone reveals that zirconia abutments offer superior aesthetic outcomes and favorable biological properties, such as reduced plaque accumulation and lower inflammatory response, making them ideal for visible areas with thin, soft tissue biotypes. Conversely, titanium abutments excel in mechanical durability, tensile strength, and fatigue resistance, ensuring long-term stability under functional loads. However, they may accumulate more plaque and incite higher inflammatory responses, posing a risk for peri-implant complications. The potential for titanium allergies, although rare, necessitates careful patient evaluation and consideration of alternatives such as zirconia. Ultimately, the choice between zirconia and titanium abutments should be tailored to the specific clinical scenario, patient needs, and aesthetic demands, with regular monitoring to ensure the success and longevity of implant restorations.
